# The risk factors for the postoperative pulmonary infection in patients with hypertensive cerebral hemorrhage: A retrospective analysis

**DOI:** 10.1097/MD.0000000000023544

**Published:** 2020-12-18

**Authors:** Shihai Xu, Bo Du, Aijun Shan, Fei Shi, Jin Wang, Manying Xie

**Affiliations:** Emergency Center, Shenzhen People's Hospital.

**Keywords:** hypertension, intracerebral hemorrhage, neurosurgery, pulmonary infection, risk

## Abstract

The risk factors for the pulmonary infections after hypertensive cerebral hemorrhage remains unclear. We aimed to investigate the potential risk factors for the postoperative pulmonary infection in patients with hypertensive cerebral hemorrhage.

Patients with hypertensive cerebral hemorrhage undergone surgery from January 2018 to December 2019 were included. Related personal and medical information were collected. Univariate and multivariate logistic regression analyses were performed to identify the potential risk factors for the postoperative pulmonary infection.

A total of 264 patients were included, and the incidence of pulmonary infection for patients with hypertensive cerebral hemorrhage after surgery was 19.70%. Escherichia coli is the most common bacteria of pulmonary infection. Multivariate regression analysis revealed that the preoperative hypoalbuminemia (OR2.89, 1.67∼4.78), tracheotomy (OR5.31, 1.24∼11.79), diabetes (OR4.92, 1.32∼9.80), preoperative GCS (OR5.66, 2.84∼11.21), and the duration of mechanical ventilation (OR2.78, 2.32∼3.61) were the independent risk factors for the pulmonary infection in patients with hypertensive cerebral hemorrhage (all *P* < .05).

Patients with hypertensive intracerebral hemorrhage after surgery have a higher risk of postoperative pulmonary infections, and there are many related risk factors, which should be taken seriously in clinical practice.

## 1 Introduction

With the changes in the style of modern life, the incidence of hypertension is on the rise, and long-term hypertension will accelerate the atherosclerosis process, posing a great threat to the cardiovascular system.^[1,2]^ Hypertensive cerebral hemorrhage is a common complication of hypertension.^[3]^ It has the characteristics of sudden onset, rapid development, and many adverse complications.^[4]^ It is been found^[5]^ that it will reduce the oxygen supply of brain tissues and cause different degrees of damage to patients neurological functions. Reaching the indication for surgery after intracerebral hemorrhage requires surgical treatment. The purpose of surgical treatment is to remove intracranial hematoma, reduce intracranial pressure, and avoid the occurrence of cerebral hernia.^[6]^ However, intracranial surgery often requires the indwelling of various drainage tubes, and the risk of complications such as rebleeding and pulmonary infection is high.^[7,8]^ Therefore, the prevention and treatment of related complications after intracranial surgery is essential.

Pulmonary infection is one of the common complications of hypertensive cerebral hemorrhage, which will aggravate brain tissue hypoxia and cause second injury to brain, and even posing a serious threat to the life and health of patients.^[9,10]^ Therefore, the prevention of pulmonary infection after surgery in patients with hypertensive cerebral hemorrhage is vital to the prognosis of patients. Currently, the risk factors for the pulmonary infections after hypertensive cerebral hemorrhage remains unclear, and understanding this do has positive significance for reducing the risk of related complications. Therefore, we conducted this study with attempt to investigate the potential risk factors for the postoperative pulmonary infection in patients with hypertensive cerebral hemorrhage, to providing insights into the treatment and management of hypertensive cerebral hemorrhage

## 2 Methods

### 2.1 Ethical consideration

This present study has been certified and approved by the ethics committee of Shenzhen People's Hospital (SX 20170086), and verbal informed consents had obtained from the included participants.

### 2.2 Participants

Patients with hypertensive cerebral hemorrhage undergone surgery in the department of neurosurgery of our hospital from January 2018 to December 2019 were included in this present study. The inclusion criteria were:

1. It was the first acute cerebral hemorrhage for patient, and the diagnosis complied with the related guidelines.^[11,12]^
2. The Glasgow coma score (GCS) at admission was between 3 and 12 points.
3. The patient showed no signs of lung infection or pneumonia at the time of admission.
4. After admission, surgery with hematoma removal or decompression of bone flaps was performed under general anesthesia for patients, and those with hematoma ruptured into the ventricle also underwent external drainage.

The exclusion criteria were:

1. patients with congenital hematological disorders.
2. The cerebral hemorrhage was caused by the malformations of cerebral vascular.

### 2.3 The diagnosis criteria for pulmonary infection

The diagnostic criteria for lung infections were based on the diagnostic standards.^[13]^ if patient showed any 3 of the following 5 items, the diagnosis of pulmonary infection can be determined:

1. respiratory symptoms such as cough, purulent sputum, and deep breathing
2. Auscultation showed both lungs with dry and wet rales and/or signs of lung consolidation in varying degrees;
3. Body temperature ≥38.5°C with an increase in peripheral white blood cell count ≥1.0 × 10^9^/L;
4. X-ray chest radiograph showed inflammatory changes;
5. Pathogens were obtained from sputum or blood culture.

### 2.4 Data collections

The included patients with hypertensive cerebral hemorrhage after surgery were divided into infection group and no infection group according to the presence or absence of concurrent pulmonary infections. Following information and data were collected and analyzed: the age; gender; the concurrent diseases such as diabetes, hyperlipidemia et al; whether or not performed the tracheotomy; the hemorrhage localization; the preoperative GCS; the duration of operations; the duration of nasal feeding tube; length of hospital stay.

### 2.5 Statistical analysis

We processed the collected data with SPASS23.0 software. Categorical variables were analyzed using the χ^2^ test or Fishers exact test, and continuous variables were analyzed using Student *t* test or Mann–Whitney *U* test, and were generally presented as means and standard deviation. Multivariate logistic regression analyses were performed using the forward likelihood ratio selection method to identify independent risk factors and it is presented with an odds ratio (OR) and 95% confidence intervals (95%CI). Potential candidate variables were those with *P* < .05 in univariate analyses. And *P* < .05 was considered statistically significant in this present study.

### 2.6 The characteristics of included patients

A total of 264 patients with hypertensive cerebral hemorrhage were included in this present study, with 52 patients in the pulmonary infection group, and 212 patients in the no infection group. The incidence of pulmonary infection for patients with hypertensive cerebral hemorrhage after surgery was 19.70%. And 12 patients had ventilator associated pneumonia. The overall average age of included patients was (66.3 ± 8.71) year old. The characteristics of included patients in infection and no infection group were shown in Table 1. There were significant differences in the diabetes, preoperative hypoalbuminemia, the duration of mechanical ventilation, preoperative GCS, COPD, tracheotomy, length of operation, the duration of nasal feeding tube among infection and no infection group (all *P* < .05), no significant differences were detected in gender, age, hyperlipidemia, hemorrhage localization and length of hospital stay(all *P* > .05).

**Table 1:** The characteristics and clinical information of included patients.

Items	Infection (n = 52)	No infection (n = 212)	χ^2^/t	*P*
Age (years)	46.5 ± 8.35	46.2 ± 9.73	11.351	.085
Gender				
Male	38	169	1.044	.105
Female	13	43		
Diabetes				
Yes	22	46	3.289	.043
No	30	166		
Hyperlipidemia				
Yes	16	39	1.537	.061
No	36	173		
Preoperative hypoalbuminemia				
Yes	42	68	1.979	.001
No	10	144		
Hemorrhage localization				
Basal ganglia	20	87	5.484	.122
Thalamus	13	51		
Cerebellum	9	40		
Ventricle	7	23		
Cerebral lobe	3	11		
The duration of mechanical ventilation (days)	2.3 ± 1.10	1.5 ± 0.85	8.250	.037
Preoperative GCS score	6.8 ± 2.64	8.7 ± 3.29	11.073	.009
COPD				
Yes	29	63	4.527	.002
No	23	149		
Tracheotomy				
Yes	30	47	2.185	.011
No	22	165		
Length of operation (min)				
<60	39	179	22.063	.009
≥60	13	33		
The duration of nasal feeding tube (days)	3.9 ± 1.01	2.1 ± 0.93	1.425	.025
Length of hospital stay (days)	5.21 ± 1.28	5.02 ± 1.14	1.755	.106

**Table 2:** Multivariate regression analysis on the risk factors of pulmonary infection in patients with hypertensive cerebral hemorrhage.

Factors	β	S̄x	OR	95%CI	*P*	Rank
Preoperative hypoalbuminemia	0.06	0.279	2.89	1.67∼4.78	.002	1
Tracheotomy	0.90	0.346	5.31	1.24∼11.79	.035	2
Diabetes	1.03	0.445	4.92	1.32∼9.80	.032	3
Preoperative GCS	0.97	0.325	5.66	2.84∼11.21	.039	4
The duration of mechanical ventilation	1.13	0.117	2.78	2.32–3.61	.045	5

### 2.7 Bacterial species distribution

**Figure 1:**
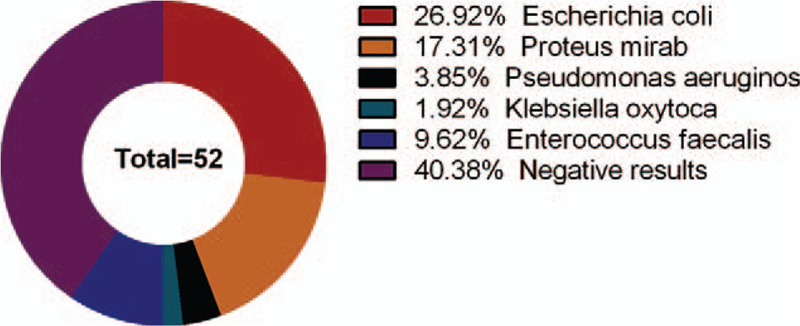
The bacterial distribution of blood culture of 52 patients with pulmonary infection.

The blood culture results of 52 infection patients indicated that there were 14 cases of Escherichia coli, 9 cases of Proteus mirabilis, 2 case of Pseudomonas aeruginosa, 1 case of Klebsiella oxytoca, 5 cases of Enterococcus faecalis, and 21 cases of negative results (Fig. 1).

**Figure 2:**
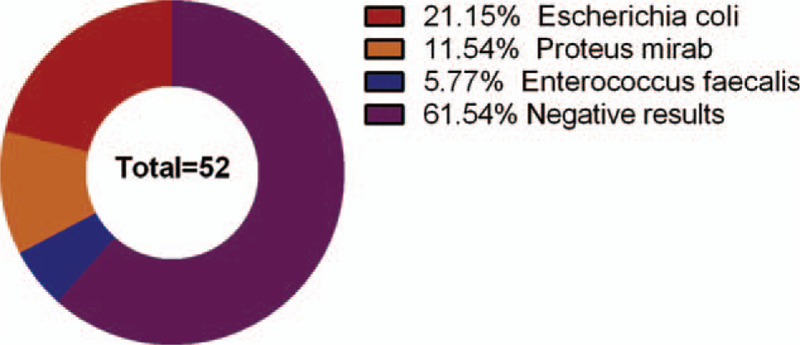
The bacterial distribution of sputum culture of 52 patients with pulmonary infection.

The sputum culture results of 52 fever patients indicated that there were 11 cases of Escherichia coli, 6 cases of Proteus mirabilis, 3 cases of Enterococcus faecalis and 32 cases of negative results (Fig. 2).

### 2.8 Multivariate regression analysis on the potential risk factors

We further performed the multivariate regression analysis on the following items with statistical significance in the univariate analysis: diabetes, preoperative hypoalbuminemia, the duration of mechanical ventilation, preoperative GCS, COPD, tracheotomy, length of operation, the duration of nasal feeding tube. And the results of multivariate regression analysis indicated the preoperative hypoalbuminemia (OR2.89, 1.67∼4.78), tracheotomy (OR5.31, 1.24∼11.79), diabetes (OR4.92, 1.32∼9.80), preoperative GCS (OR5.66, 2.84∼11.21), and the duration of mechanical ventilation (OR2.78,2.32∼3.61) were the independent risk factors for the pulmonary infection in patients with hypertensive cerebral hemorrhage (all *P* < .05).

## 3 Discussion

Pulmonary infection is a common complication after intracerebral hemorrhage. The incidence of pulmonary infection after intracerebral hemorrhage in this present study is 19.70%. It is been reported that the incidence of pulmonary infection in conservatively treated patients after intracerebral hemorrhage is 18%,^[14]^ which is lower than that of our findings. It is understandable that postoperative patients are more likely to have pulmonary infections, the patients who have reached the surgical indication generally have larger hematomas and deeper injury of brain functions.^[15]^ Furthermore, patients may need to retain various ducts after surgery to maintain the intracranial pressure in normal scope.^[16,17]^ Therefore, postoperative patients are more likely to have pulmonary infections than those patients that did not undergo surgery treatment. Furthermore, our results have indicated that the preoperative hypoalbuminemia, tracheotomy, diabetes, preoperative GCS, and the duration of mechanical ventilation were the independent risk factors for the pulmonary infection in patients with hypertensive cerebral hemorrhage, more attentions should be paid and prophylaxis must be conducted for those patients.

In this study, gram-negative bacteria has accounted for the majority of bacterial infections. This may be caused by long-term resident bacteria in the lungs caused by secretion accumulations.^[18]^ It may be related to bacterial imbalance caused by long-term use of antibacterial drugs.^[19]^ Gram-positive bacteria mainly cause lung infection through invasion through invasive procedures and surgery.^[20]^ The types of pathogenic bacteria in patients with hypertensive intracerebral hemorrhage combined with pulmonary infection are more complicated.^[21]^ Therefore, accurate drug susceptibility identification has guiding significance in selecting the appropriate antibacterial drug. It is been reported^[22–24]^ that Staphylococcus and Staphylococcus epidermidis are sensitive to vancomycin; Gram-negative bacteria Klebsiella pneumoniae and Acinetobacter baumannii are sensitive to cefoperazone. Antibiotics should be selected clinically based on the results of drug sensitivity tests.^[25]^

Patients with hypertensive intracerebral hemorrhage are in a state of high consumption and prone to hypoalbuminemia when the nutritional supply is not enough. Patients with hypoalbuminemia are prone to be infected due to decreased albumin and globulin.^[26,27]^ At the same time, albumin is an important part of colloid osmotic pressure in blood vessels. Hypoalbuminemia can cause changes in colloid osmotic pressure and make extracellular fluid hypotonic, which can cause hypotonic dehydration and electrolyte disorders.^[28,29]^ The results of this study have showed that the patients with hypoalbuminemia is more likely to get pulmonary infection than the patients with normal albumin. Therefore, patients with intracerebral hemorrhage should be supplemented with sufficient nutrients, and monitor the changes in albumins.^[30]^ If necessary, supplement human albumin to keep the balance.

Tracheostomy is an important risk factor of pulmonary infection for patients with hypertensive cerebral hemorrhage.^[31]^ In patients with severe conscious disturbance, the large amount of sputum in the lungs cannot be discharged spontaneously, tracheostomy may be performed to keep the airway open.^[32]^ However, there are many disadvantages related to tracheotomy. For example, due to the change of natural breathing passages, the inhaled air lacks filtration and humidification of the nasal mucosa, bacteria may easily invade, it is prone to be infected.^[33]^ Therefore, patients with tracheotomy should be given nebulization and moistened inhaled air, and those with thick sputum should be given suction treatment.^[32]^ The sputum suction should be gentle to reduce the irritation of the tracheobronchial mucosa. If necessary, relevant antibiotics may be used for anti-infective treatment.^[34]^

In patients with diabetes, due to abnormal glucose metabolism, the bodys energy supply is unbalanced, and the immunity is reduced, thus infection is more likely to occur.^[35]^ Previous studies^[36]^ have shown that people with poor glycemic control are at higher risk of infection. GCS is an important indicator of the functions of the central nervous system. The hematomas formed after hypertensive intracerebral hemorrhage compress the adjacent nerve tissues and blood vessels, causing cerebral tissue ischemia and hypoxia, affecting the blood supply and metabolism of nervous tissues, and edema caused by hematoma aggravated the degree of cerebral ischemia and hypoxia, the occupation of hematoma and edema can cause intracranial hypertension.^[37,38]^ With the impairment of patients consciousness, and the GCS decrease accordingly, and the patients cough reflex and swallowing function were impaired, which easily caused aspiration and sputum could not be excreted timely, thus increasing the chance of lung infection. Therefore, for patients with severe conscious disorders, intensify respiratory care after surgery are highlighted.

The duration of mechanical ventilation is also an important risk factor. Tracheal intubation can directly damage the tracheal mucosa, leading to a decrease in the defense capacity of airway.^[39]^ At the same time, the oropharyngeal bacteria are directly brought into the airway during intubation, which increases the probability of lung infection.^[40]^ Besides, repeated suctioning can also directly damage the tracheal mucosa. Tracheal intubation changes the natural airway, avoids the filtering of air by the nasal mucosa, increases the chance of bacteria entering the lungs.^[41]^ It is been reported^[42]^ that the sputum in the gap between the balloon and the glottis cannot be eliminated by suction, and it is easy to breed bacteria, leading to that biofilm attach to the tracheal tube. These bacteria can migrate down the respiratory tract, increase the chance of pulmonary infections. Previous studies^[43,44]^ have confirmed that the subglottic secretion drainage is beneficial to combating the pulmonary infection. Study^[45]^ has shown that pulmonary infections are prone to occur after mechanical ventilation, mainly because the bacteria that adhere to the upper respiratory tract and it can be blown to the lower respiratory tract by tracheal tube. When using the ventilator, it should be noted that the ventilator tube should be replaced and disinfected regularly to avoid breeding bacteria, and the gas should be completely humidified, this will reduce the bacterial blow-in and airway mucosal damage to a certain extent, thereby reducing incidence of pulmonary infections.^[46]^

It must be aware that there are several other factors may also be associated with the pulmonary infections in patients with hypertensive intracerebral hemorrhage. Patients with hypertensive intracerebral hemorrhage need to insert nasal feeding tubes for feeding to ensure enteral nutrition due to unconsciousness or difficulty in swallowing. Indwelling nasal feeding tubes for too long can increase the incidence of pneumonia.^[47]^ We could not include those information for analysis with concerns that the collected records were incomplete. Furthermore, preoperative vomiting may be associated with the aspiration pneumonia.^[48]^ Patients with cerebral hemorrhage vomit due to high intracranial pressure caused by hematoma and edema. And the decrease in cough reflex and swallowing function caused by damage of the central nervous system, the vomitus is easily sucked into the trachea in the oropharynx, causing aspiration related pneumonia. All those potential risks merits to be considered in the future.

Several limitations in this study must be considered. Only patients that underwent surgery. Firstly, we only included the patients that underwent surgery with consideration that it may contribute to great heterogeneity if we have included all the ICH patients, but several patients with large ICH are medically managed and have a similar length of stay. Secondly, the sample size of our study was small, future studies with larger sample size and multi-centers are needed in the future.

## 4 Conclusions

In conclusion, the incidence of postoperative pulmonary infection in patients with hypertensive cerebral hemorrhage is rather high, and the preoperative hypoalbuminemia, tracheotomy, diabetes, preoperative GCS, and the duration of mechanical ventilation were the independent risk factors for the pulmonary infection in patients with hypertensive cerebral hemorrhage. Corresponding preventive and therapeutic measures should be taken clinically based on these risk factors. At the same time, given the relatively small sample size and limited data collected in this study, future studies with larger sample are needed to further explore its related risk factors, thus providing evidences for the treatment of hypertensive cerebral hemorrhage.

## Author contributions

S X, M X designed research; S X, B D, A S conducted research; S X, F S analyzed data; S X and ZJ W wrote the first draft of manuscript; M X had primary responsibility for final content. All authors read and approved the final manuscript.

**Conceptualization:** Shihai Xu, Fei Shi.

**Formal analysis:** Shihai Xu, Aijun Shan, Jin Wang, Manying Xie.

**Investigation:** Shihai Xu, Bo Du, Aijun Shan, Fei Shi, Jin Wang.

**Methodology:** Aijun Shan.

**Project administration:** Shihai Xu, Bo Du.

**Resources:** Shihai Xu, Fei Shi, Jin Wang, Manying Xie.

**Software:** Shihai Xu, Bo Du, Jin Wang, Manying Xie.

**Supervision:** Shihai Xu, Bo Du, Manying Xie.

**Validation:** Bo Du, Manying Xie.

**Visualization:** Bo Du.
